# *Ramie leaf* Extract Alleviates Bone Loss in Ovariectomized Rats—The Involvement of ROS and Its Associated Signalings

**DOI:** 10.3390/nu15030745

**Published:** 2023-02-01

**Authors:** Geum-Hwa Lee, The-Hiep Hoang, Hwa-Young Lee, Young-Je Lim, Ji-Hyun Kim, Su-Jin Jung, Soo-Wan Chae, Mohammad Mamun Ur Rashid, Han-Jung Chae, Sun-Jung Yoon

**Affiliations:** 1 Research Institute of Clinical Medicine of Jeonbuk National University-Biomedical Research Institute of Jeonbuk National University Hospital, Jeonju 54907, Republic of Korea; 2 Department of Obstetrics and Gynecology, Hue University of Medicine and Pharmacy, Hue University, Hue 52000, Vietnam; 3 Non-Clinical Evaluation Center, Biomedical Research Institute, Jeonbuk National University Hospital, Jeonju 54907, Republic of Korea; 4 Clinical Trial Center for Functional Foods (CTCF2), Jeonbuk National University Hospital, Jeonju 54907, Republic of Korea; 5 Department of Pharmacology, Jeonbuk National University Medical School, Jeonju 54896, Republic of Korea; 6 School of Pharmacy, Jeonbuk National University, Jeonju 54896, Republic of Korea; 7 Department of Orthopedic Surgery, Jeonbuk National University Medical School, Jeonju 54907, Republic of Korea

**Keywords:** *Ramie leaf*, RANKL, osteoporosis, ROS, osteoclastogenesis

## Abstract

*Ramie leaf* (*Boehmeria nivea* L.) has been traditionally used to treat gynecological and bone-related disorders. This study aims to evaluate the effect of *Ramie leaf* extracts (RLE) against osteoporosis in ovariectomized (OVX) rats. Female SD rats aged seven weeks were randomly assigned into five OVX and a sham-operated (sham) group. OVX subgroups include OVX, vehicle-treated OVX group; E2, OVX with 100 μg/kg 17β-estradiol; and RLE 0.25, 0.5, and 1, OVX rats treated with 0.25, 0.5, and 1 g/kg/day RLE, respectively. Two weeks into the bilateral ovariectomy, all the rats were orally administered with or without RLE daily for 12 weeks. OVX rats administered with RLE showed higher bone density, relatively low tartrate-resistant acid phosphatase (TRAP)-positive osteoclasts, and lower reactive oxygen species (ROS) within bone tissues compared to vehicle-treated OVX rats. Furthermore, supplementation of RLE improved bone mineral density (BMD) and bone microstructure in the total femur. RLE prevented RANKL-induced osteoclast differentiation and expression of osteoclastogenesis-related genes such as Cal-R, MMP-9, cathepsin K, and TRAP in RANKL-induced RAW264.7 cells. Moreover, RLE administration lowered the intracellular ROS levels by reducing NADPH oxidase 1 (NOX-1) and 4-hydroxynonenal (4HNE). These results suggest that RLE alleviates bone mass loss in the OVX rats by inhibiting osteoclastogenesis, where reduced ROS and its associated signalings were involved.

## 1. Introduction

Osteoporosis or bone loss is often considered the most widespread metabolic bone disorder affecting humans and represents a major public health concern [1,2]. Specifically, in women, reduced estrogen levels following menopause are a well-known risk factor for osteoporosis. Osteoporosis is often treatable with medications. However, side effects such as gastrointestinal disorders, esophagitis, myalgia, or atypical fracture [3] encourage patients to consider alternative treatments. Traditionally, denosumab and bisphosphonates are used to treat osteoporosis, but their long-term consumption may increase the risk of cancer and osteonecrosis [4]. With these concerns, substantial research was conducted on natural medications [5] with little success. Ramie (*Boehmeria nivea* L.) is a flowering plant from the Urticaceae family. It is native to Southeast Asia and abundantly available in Korea, the Philippines, and China [6,7]. Traditionally, ramie leaves are used to prepare tea and rice cakes in Korea. Ramie is a good source of protein, fatty acids, vitamins, and minerals. *Ramie leaf* extracts have superior physiological functions, including antioxidant, antifungal, and antibacterial properties that suppress bacterial and fungal infections. Additionally, it has demonstrated anticancer properties against lung and liver cancer. Further, *Ramie leaf* extracts are rich in phytochemicals such as flavonoids and phenolics, which have dietary and therapeutic benefits. These characteristic properties of *Ramie leaf* extract exert higher antioxidant activity than a typical diet. Moreover, ramie leaves contain various bioactive compounds such as flavonoids and polyphenols [8,9]. Hence, *Ramie leaf* extract is a functional food that is nutritionally rich and offers several health benefits.

Previous investigations indicate that bone pathogenesis, osteoporosis, bone tumor development, diabetes-induced bone complications, and joint inflammatory diseases are influenced by oxidative stress [10,11]. Specifically, reactive oxygen species (ROS) play a significant role in mineral homeostasis, affecting bone remodeling by accelerating bone resorption [12,13]. In young people, osteoporotic syndromes are associated with oxidative stress, and patients with osteoporosis demonstrated lowered serum antioxidant levels [14,15]. Similarly, osteoporotic postmenopausal women have lower antioxidant levels with higher oxidative-stress-linked biomarkers. Previous investigations revealed that ROS levels potentially regulated bone resorption activity in osteoclasts [16], and its overproduction could affect nuclear factor kappa B (NF-κB)-mediated inflammation. Thus, polyphenols with antioxidative and anti-inflammatory potentials are considered a reliable and safe treatment for postmenopausal osteoporosis [17,18].

Further, nuclear factor E2-related factor 2 (Nrf-2) is crucial for maintaining oxidative equilibrium by regulating several genes involved in ROS clearance [19]. Under oxidative stress, Nrf-2 translocates to the nucleus and binds to antioxidant response elements (AREs) that stimulate the production of superoxide dismutase (SOD), glutathione peroxidase (GPx), and catalase (CAT). Supporting these observations, ovariectomized Nrf-2-knockout mice improved bone turnover, which is responsible for the deterioration of bone structure in osteoporotic mice. This suggests that transcription factor could be a promising therapeutic target for bone mass maintenance. Here, we investigated the effects of *Ramie leaf* extract (RLE) on osteoporosis using an ovariectomized rat model. Moreover, we evaluated the effect of canthaxanthin (CX), a main component of RLE. The study observations offer prospects of the therapeutic potential of RLE and reveal a mechanism that could lead to the development of a novel therapeutic option to treat osteoporosis in postmenopausal women.

## 2. Materials and Methods

### 2.1. Preparation and Analysis of Ramie leaf Extract (RLE)

RLE was prepared by steaming dried *ramie leaves*. Briefly, ramie leaves were finely ground, dried, and extracted using boiling distilled water. Extracts were then concentrated with a rotary evaporator at reduced pressure and lyophilized to produce RLE.

### 2.2. Cell Culture and Osteoclastogenesis

Murine macrophage cells (RAW 264.7) were purchased from ATCC (Manassas, VA, USA). RAW 264.7 cells were maintained in Dulbecco’s Modified Eagle Medium (DMEM) containing 10% FBS and 1% penicillin/streptomycin under standard conditions. Cells were seeded in 5 × 10^4^ cells/well in 24-well plates in the presence of RANKL (100 ng/mL; R&D Systems, Minneapolis, MN, USA, #TNSF11) and M-CSF (20 ng/mL; R&D Systems, Minneapolis, MN, USA, #416-ML). Various concentrations of RLE (25, 50, and 100 μg/mL), 100 μM NAC (Sigma-Aldrich, St. Louis, MO, USA, #7250), and 10 μg/mL CX (sigma, #11775) were added to these cultures for 7 days. The culture medium was replaced with fresh medium every 2 days. Osteoclast formation was measured using the TRAP staining kit on day 7. Briefly, adherent cells were fixed with 10% formaldehyde in PBS for 30 min, and TRAP-positive cells with more than three nuclei were scored as osteoclasts. The vitality of cells treated with RLE at concentrations of 25, 50, and 100 μg/mL and other agents was evaluated using MTT assay kit (Promega, Madison, WI, USA, #G4000) by following kit instructions.

### 2.3. HPLC (High-Performance Liquid Chromatography) Assay

Prepared RLE was analyzed with an HPLC system (Agilent 1260 infinity, Santa Clara, CA, USA) with 3.5 µm Phnomenex kinetex C8 column (Agilent, Santa Clara, CA, USA). The mobile phase contained two solvents: A, 0.1% formic acid/water; B, 0.1% formic acid in water/acetonitrile. The 5 µL injection volume was allowed to flow at a rate of 1 mL/min at a temperature of 30 °C. Canthaxanthin was procured from Sigma-Aldrich (St Louis, MO, USA) and analyzed at 490 nm. Peak analyses and assignments were performed according the UV spectra and retention times of canthaxanthin in the chromatograms. Upon HPLC analysis, canthaxanthin was identified as a component and level of 1.93 mg/g, respectively.

### 2.4. Animal Grouping and Experimental Protocol

Female (7 weeks old) Sprague–Dawley (SD) rats were purchased from Orient Science Co. (Seongnam, Korea). Rats were housed at 22 ± 2 °C with a 12 h LD cycle under 55–60% humidity in the SPF facility. Animals were provided with a standard chow diet with free access to water. All the animal procedures were performed strictly following the Jeonbuk National University Hospital Institutional Animal Care and Use Committee’s guidelines for the care and use of laboratory animals (JBUH-IACUC-2021-27). Bilateral ovariectomy (OVX) was performed under anesthesia. Rats were bilaterally ovariectomized and fostered for two weeks to allow for recovery and removal of endogenous sex hormones. A day after surgery, the OVX rats were divided into the following groups (*n* = 10 per group): one with no further treatment (OVX control group); five groups administered RLE at doses of either 0.25, 0.5, or 1 g/kg body weight (RLE, respectively); and one that received 17β-estradiol at a dose of 100 μg/kg body weight (E2 group), serving as a positive control. RLE extract and E2 were dissolved in 0.9% saline and administered orally once a day for 12 weeks. Body weight of all experimental rats were assessed once a week during the intervention period. After 12 weeks, all the rats were euthanized for the isolation of serum and biochemical analysis.

### 2.5. Tartrate-Resistant Acid Phosphatase (TRAP) Assay

RAW 264.7 cells were treated with 100 ng/mL RANKL and supplemented with different dosages of RLE for 7 days to determine the efficacy of RLE on osteoclast differentiation. Next, mature osteoclasts were washed with Dulbecco’s phosphate buffer saline (DPBS) and stained with TRAP using a commercial acid phosphatase leukocyte kit (Sigma, #387A). For cytochemical staining of TRAP-positive cells, deparaffinized tissue sections or 10% formaldehyde-fixed cells were stained for TRAP following the manufacturer’s protocol. TRAP-positive cells were counted microscopically (EVOS M7000, Thermo Fisher Scientific Inc., Waltham, MA, USA), and their activity was determined as described previously [20].

### 2.6. Immunoblotting Analysis

Immunoblotting was performed as outlined previously [21]. Briefly, cell lysates were separated by 4–12% SDS-PAGE, transferred to a PVDF membrane, blocked with 5% skim milk, and incubated overnight with the antibodies as specified. The blots were incubated with relevant primary antibodies overnight at 4 °C and with corresponding secondary antibodies. The primary antibodies used in this study are as follows: The antibodies against NFATc1 (#sc7294), c-fos (#sc271243), Nrf-2 (#sc722), and β-actin (sc47778) were purchased from Santa Cruz Biotechnology (Santa Cruz, CA, USA). The antibodies against TRAF-6 (#ab227560) and NOX-1 (#ab131088) were provided by Abcam (Cambridge, UK). Protein signal was detected using enhanced chemiluminescence reagents (Bio-Rad, Hercules, CA, USA).

### 2.7. RNA Extraction and Reverse-Transcription Polymerase Chain Reaction (RT-PCR)

RAW 264.7 cells (1 × 10^5^) were cultured in six-well plates and treated with RLE (25, 50, 100 μg/mL) or canthaxanthin (CX) in the presence of RANKL. For six days, the culture medium was switched every other day. Total RNA was extracted with Trizol (Invitrogen, Carlsbad, CA, USA, #10296028). Complementary DNA was synthesized using 1 μg extracted RNA with a reverse transcription kit (TaKaRa Bio, Otsu, Japan, #RR037A) according to the manufacturer’s guidelines. The cDNA was amplified using PCR with specific primers and thermal cycling conditions for cathepsin K, Cal-R, MMP-9, TRAP, and GAPDH. All the primers (Bioneer, Daejeon, Korea) used in this study are listed in Table 1. All the PCR were performed in triplicate. Finally, gels were imaged with *Gel Doc XR+System* (Thermo Fisher Scientific) and analyzed with Image J (National Institutes of Health, Bethesda, MD, USA).

### 2.8. Cellular ROS Detection

For ROS detection in bone tissue and Raw 264.7 cells, sections were incubated with using the CellROX^®^ Red (Thermo Fisher Scientific, Waltham, MA, USA, #C10422) or DHE Reagent (Thermo Fisher Scientific, Waltham, MA, USA, #D23806) according to the manufacturer’s recommended protocol. Images were acquired fluorescent microscope (EVOS M7000, Thermo Fisher Scientific, Waltham, MA, USA) and quantitatively analyzed using Image J (National Institutes of Health, Bethesda, MD, USA).

### 2.9. MitoSOX Staining

The fixed bone tissues were then washed with 1x phosphate-buffered saline (PBS) and incubated with 1x PBS containing 5 μM MitoSOX (Invitrogen, Waltham, MA, USA, #M36008) for 40 min at 37 °C in a dry oven. The specimens were washed with 1x PBS. The slides containing ovarian tissues were observed with a fluorescence microscope (EVOS M7000, Thermo Fisher Scientific, Waltham, MA, USA). All parts of each slide were observed, and representative images were captured and analyzed by the ImageJ program (National Institutes of Health, Bethesda, MD, USA).

### 2.10. Assessment of Bone Microstructure Using Micro-Computed Tomography

Bone microstructure was assessed with micro-computed tomography (CT). A 3D-microCT of freshly isolated fourth lumbar vertebrae was performed as described previously [18] using micro-CT (Skyscan 1076, Skyscan Co., Antwerp, Belgium). Each sample was analyzed for trabecular number (Tb.N), trabecular thickness (Tb.Th), bone volume to tissue volume (BV/TV), and trabecular separation (Tb.Sp) in the metaphysis area calculated using CT analysis software.

### 2.11. Serum Parameters

To determine bone resorption, β-C-terminal telopeptide of type 1 collagen (CTx-1) was examined using rat ELISA kits (MyBioSource, Inc., San Diego, CA, USA, #MBS703743). Similarly, levels of osteocalcin (OC, #MBS2022619) and ALP were determined using relevant rat ELISA kits (MyBioSource, San Diego, CA, USA, #MBS011598).

### 2.12. Immunohistochemical (IHC) Staining

Immunohistochemical staining was performed as described earlier [18]. The left femur was fixed, demineralized, embedded in paraffin, and appropriately sectioned. Then, sections were deparaffinized with xylene, rehydrated in gradient alcohol, and stained with hematoxylin and eosin (H&E). For IHC staining, section slides were incubated overnight at 4 °C with primary antibodies against NOX-1 (abcam, #ab131088), 4HNE (Santa Cruz, #sc130083), cathepsin K (Santa Cruz, #sc48353), or NFATc1(Santa Cruz, #sc7294) and then with HRP-conjugated secondary antibodies. Next, endogenous peroxide was blocked with 3% hydrogen peroxide and incubated with EnVision+ System-HRP (DAKO, Glostrup, Denmark, #K4065). For visual analysis, the product was stained with diaminobenzidine (DAB) and counterstained with Mayer’s hematoxylin. All the acquired images were assessed with Image J (National Institutes of Health, Bethesda, MD, USA).

### 2.13. Statistical Analysis

All the data were analyzed using GraphPad Prism v8.0 (GraphPad Software, San Diego, CA, USA). One-way ANOVA with Tukey’s post hoc test was done for multiple comparisons. Data are expressed as the mean ± SEM. *p <* 0.05 indicates the statistical significance.

## 3. Results

### 3.1. Analysis of Compounds in RLE Extract

**Figure 1:**
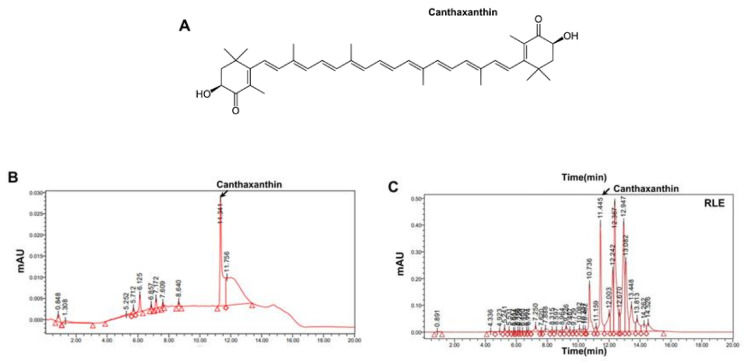
HPLC analysis of *Ramie leaf* extracts. (**A**) Chemical structure of canthaxanthin (CX). (**B**) Chromatograms of CX standard (**C**) and RLE analyzed for CX.

Canthaxanthin (CX), a carotenoids compound majorly found in *Ramie leaf* (RL), was quantified with HPLC. Canthaxanthin is known as six major botanical carotenoids together with β-carotene, lycopene, lutein, zeaxanthin, and α-carotene [22]. Figure 1A shows the structure of CX. HPLC chromatogram regarding CX standard and RLE is representatively shown (Figure 1B,C).

### 3.2. Effects of RLE on Body and Uterine Weight in OVX Rats

**Figure 2:**
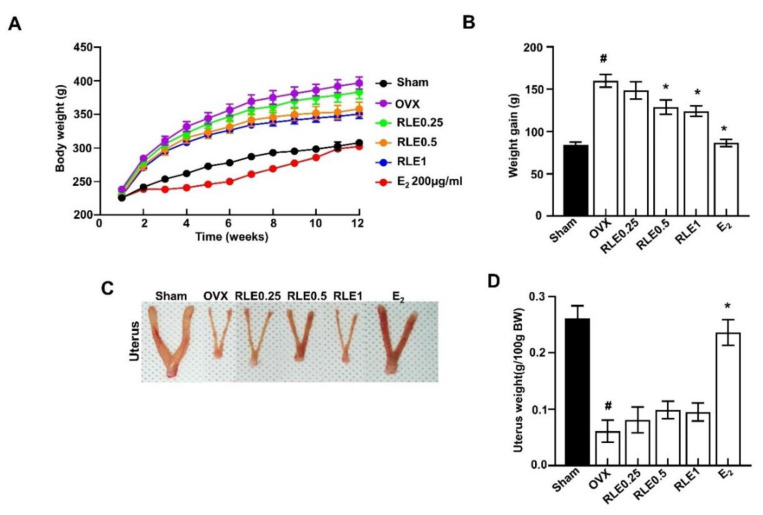
Influence of RLE on the body and uterus weight. (**A**) Body weight and (**B**) weight gain. (**C**) Representative uterus image and (**D**) uterus weight. Data are shown as mean ± SEM (*n* = 10, ^#^ *p* < 0.05 vs. sham; * *p* < 0.05 vs. OVX). Sham, sham-operated group; OVX, ovariectomy; RLE, *Ramie leaf* extract.

The OVX group had significantly higher body weight than the sham group. However, RLE administration prevented the increase in body weight dose-dependently (Figure 2A,B). These results demonstrate that RLE reduced the OVX-induced weight gain. Contrastingly, the OVX group had significantly less uterine weight than the sham group. However, different concentrations of RLE had no impact on uterine mass (Figure 2C,D).

### 3.3. Effect of RLE against Bone Mass Loss in the OVX Rats

**Figure 3:**
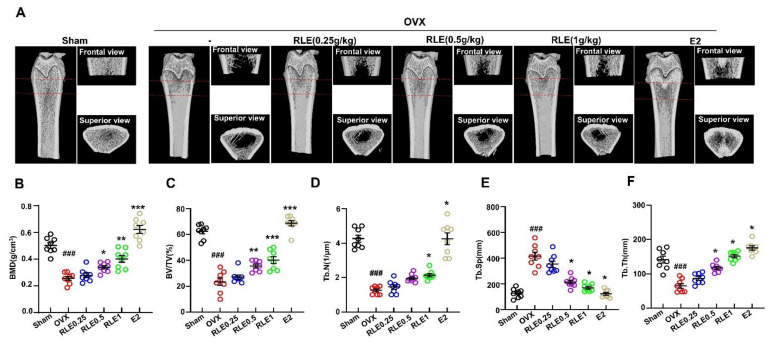
RLE prevents OVX-induced bone mass loss in rats. (**A**) Representative microCT images of the femur bone. (**B**–**F**) Bone structural characteristics such as bone mass density (BMD in g/cm^3^), ratio of bone volume to total volume (BV/TV in %), trabecular number (Tb.N in 1/mm^2^), Tb.Sp (mm), and trabecular thickness (Tb.Th) are shown. Data are shown as mean ± SEM. (*n* = 10, ^###^ *p* < 0.001 vs. sham; * *p* < 0.05 vs. OVX, ** *p* < 0.01 vs. OVX, *** *p* < 0.001 vs. OVX). Sham, sham-operated group; OVX, ovariectomy; RLE, *Ramie leaf* extract.

Next, 3D-microCT was performed to determine the bone characteristic features in all the groups. Together, the observations revealed that the OVX group underwent greater bone mass loss than the sham group. However, RLE administration significantly regulated the loss (Figure 3A). Next, bone structural parameters such as bone mineral density (BMD), BV/TV, Tb.N, Tb.Sp, and Tb.Th were compared between OVX and OVX + RLE groups. RLE administration alleviated these bone structural characteristics dose-dependently (Figure 3B–F).

### 3.4. Effect of RLE on Biochemical Parameters in OVX Rats

**Figure 4:**
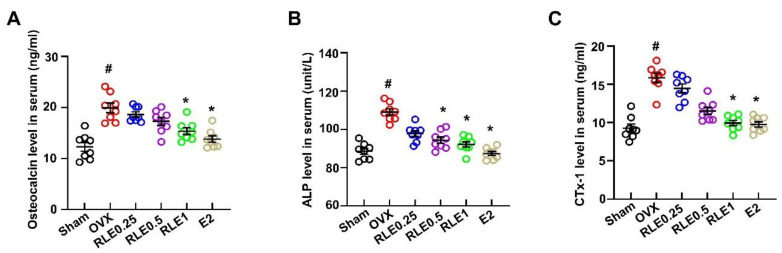
RLE regulates serum biochemical markers in OVX rats. (**A**) Osteocalcin, (**B**) ALP, and (**C**) CTx-1 expression levels in serum were analyzed by ELISA. Data are shown as mean ± SEM. (*n* = 10, ^#^ *p* < 0.05 vs. sham; * *p* < 0.05 vs. OVX). Sham, sham-operated group; OVX, ovariectomy; RLE, *Ramie leaf* extract; E2, 17β-estradiol; CTx-1, β-C-terminal telopeptide of type 1 collagen; ALP, alkaline phosphatase.

Bone turnover markers such as osteocalcin, alkaline phosphatase (ALP), osteocalcin, and CTx-1 were analyzed to evaluate the effects of RLE in OVX-induced osteoporosis. OVX increased the levels of osteocalcin, ALP, and CTx-1, while RLE and E2 treatment significantly inhibited the increased level (Figure 4A–C).

### 3.5. Effect of RLE against Trabecular Bone Loss and Osteoclastogenesis

**Figure 5:**
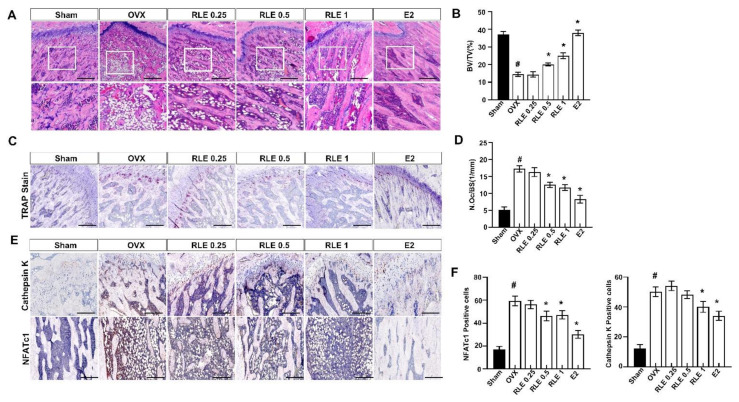
RLE regulates trabecular bone loss in OVX-induced osteoporosis model. H&E and TRAP staining of distal femoral metaphysis regions. (**A**) Representative H&E stained femur bone sections (scale bars is 200 μm). (**B**) The analysis of BV/TV based on the H&E stained femur bone sections. (**C**) Representative TRAP-stained femur bone. Image J was used to estimate trabecular area and (**D**) osteoclast number per bone surface. (**E**) Representative NFATc1 and cathepsin-K-stained femur bone. (**F**) NFATc1 and cathepsin-K-positive cells were counted and quantified relatively. Data are shown as mean ± SEM. (*n* = 10, ^#^ *p* < 0.05 vs. sham; * *p* < 0.05 vs. OVX). Sham, sham-operated group; OVX, ovariectomy; RLE, *Ramie leaf* extract; TRAP, tartrate-resistant acid phosphatase; N.oc, osteoclast numbers per bone surface.

Femur bones were stained with H&E and TRAP to evaluate the effect of RLE on bone loss and osteoclast numbers. OVX reduced the trabecular area in the femoral bone, whereas RLE significantly recovered the OVX-induced bone mass loss (Figure 5A). Additionally, BV/TV (%) was observed to be significantly lower in the OVX group compared to the sham group, whereas RLE administration improved BV/TV dose-dependently (Figure 5B). These observations indicate the potential role of RLE against bone mass loss in OVX-induced osteoporosis rats. Since multiple investigations indicated heightened osteoclast activity in the pathogenesis related to osteoporosis [17], the number of osteoclasts were measured in OVX-induced osteoporosis rats. TRAP staining of bone sections showed that the OVX group had significantly higher numbers of osteoclasts than the sham group (Figure 5C). Further, RLE administration significantly reduced the number of osteoclasts per bone surface (Figure 5D). Immunohistochemistry (IHC) analysis revealed significantly higher number of cathepsin K and NFATc1-positive cells in the OVX group. However, RLE supplementation inhibited the number of cathepsin-K- and NFATc1-positive cells dose-dependently (Figure 5E,F). Together, these observations strongly demonstrate the potential of RLE in regulating bone loss by reducing the growth of TRAP-positive osteoclasts and preventing osteoclast bone resorption activity.

### 3.6. Effects of RLE on ROS-Mediated Signaling in OVX Rats

**Figure 6:**
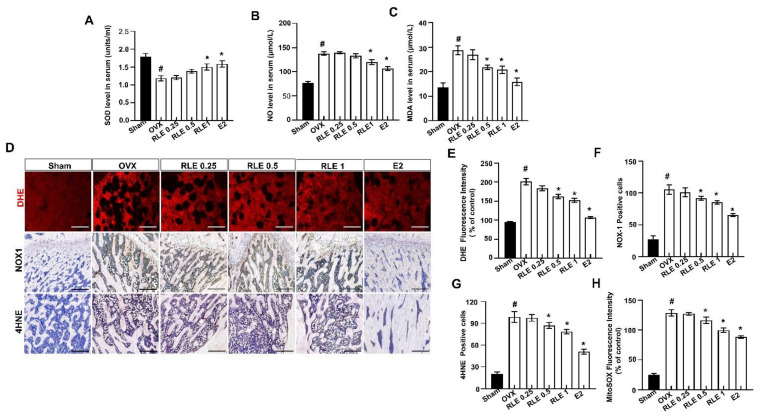
RLE regulates production of ROS in the bone tissues of the OVX rats. (**A**) SOD, (**B**) NO, and (**C**) MDA levels in rat serum. (**D**) Images of immunofluorescence staining DHE. Representative femur sections for NOX-1 and 4HNE by immunohistochemistry (IHC). (**E**) Quantitative analysis of DHE fluorescence intensity. (**F**) NOX-1- and (**G**) 4HNE-positive cells were counted and quantified relatively. (**H**) Quantitative analysis of MitoSOX fluorescence intensity. Data are shown as mean ± SEM. (*n* = 10, ^#^ *p* < 0.05 vs. sham; * *p* < 0.05 vs. OVX). Sham, sham-operated group; OVX, ovariectomy; RLE, *Ramie leaf* extract; SOD, superoxide dismutase; NO, nitric oxide; MDA, malondialdehyde, 4HNE; 4-hydroxynonenal.

Elevated ROS levels and activation of redox signaling pathways influence osteoclast differentiation [23]. OVX-induced oxidative stress, SOD, NO, and MDA levels in the rat serum were determined to confirm the bone protective effects of RLE. SOD levels were significantly lower in the OVX group than in the sham group, whereas NO and MDA levels increased. However, RLE administration significantly recovered the SOD and regulated both NO and MDA (Figure 5A–C). Further, ROS levels were measured with a DHE stain, where RLE was observed to inhibit ROS (Figure 6D,E). Furthermore, immunohistochemistry analysis revealed that NOX-1 (NADPH oxidase 1)- and 4-hydroxynonenal (4HNE)-positive cells were greatly enhanced in the bone tissues of the OVX group, while RLE administration effectively regulated the NOX-1 and 4HNE (Figure 6F,G). Similarly, RLE administration inhibited superoxide production in mitochondria (Figure 6H).

### 3.7. Effects of RLE on RANKL-Induced Osteoclastogenesis

**Figure 7:**
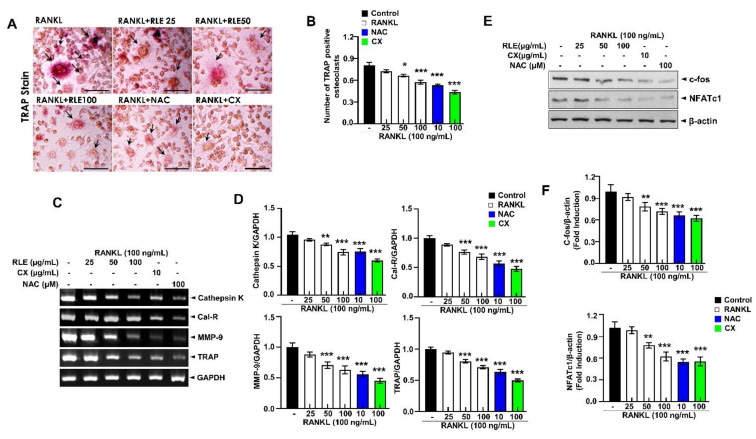
RLE inhibits the RNAKL-induced osteoclast formation in RAW264.7 cells. (**A**) Representative TRAP-stained images (Scale bar = 200 μm). (**B**) Quantitative analysis of TRAP-positive cells. (**C**) Expression of osteoclast-specific genes measured by RT-PCR. (**D**) Quantification of gene expression. (**E**) Immunoblotting using antibodies against NFATc1, c-fos, and β-actin and (**F**) respective quantification analysis. Data are shown as mean ± SEM. (*n* = 3, * p < 0.05, ** p < 0.01, *** p < 0.001 vs. RANKL) RLE, *Ramie leaf* extract; NAC, N-acetyl cysteine; MMP, matrix metallopeptidase; TRAP, tartrate-resistant acid phosphatase; cal-R, Calreticulin.

In order to determine the RLE-induced regulating effect on RANKL-induced osteoclast formation, nontoxic concentrations of RLE (0, 25, 50, or 100 μg/mL) were used to treat RAW 264.7 cells. TRAP staining showed that the control group had numerous osteoclasts and also had large osteoclasts, but the RLE-administered group had significantly lower and smaller osteoclasts, which suggests the greater influence of RLE on osteoclast formation (Figure 7A,B). Next, osteoclast-specific genes such as Cal-R, cathepsin K, TRAP, and MMP-9 were determined to evaluate the effects of RLE. These osteoclast-specific genes play a crucial role in osteoclast function and differentiation. RT-PCR analysis showed that RLE dose-dependently downregulated mRNA levels of osteoclast-specific genes (Figure 7C,D). These observations indicated the influence of RLE in inhibiting the expression of osteoclast-specific genes, which regulate the function and differentiation of osteoclasts. NFATc1 and c-fos are essential regulators to initiate osteoclast differentiation [17]. Thus, the NFATc1 and c-fos protein levels were determined to define the impact of RLE. Immunoblot observations confirm a significant decrease in NFATc1 and c-fos protein levels following RLE treatment (Figure 7E,F). These results indicate that RLE potentially inhibits osteoclastogenesis.

### 3.8. RLE Suppresses RANKL-Induced ROS Production and Its Associated Signalings, TRAF-6, NOX-1, Nrf-2, and HO-1 Pathways, during Osteoclastogenesis

**Figure 8:**
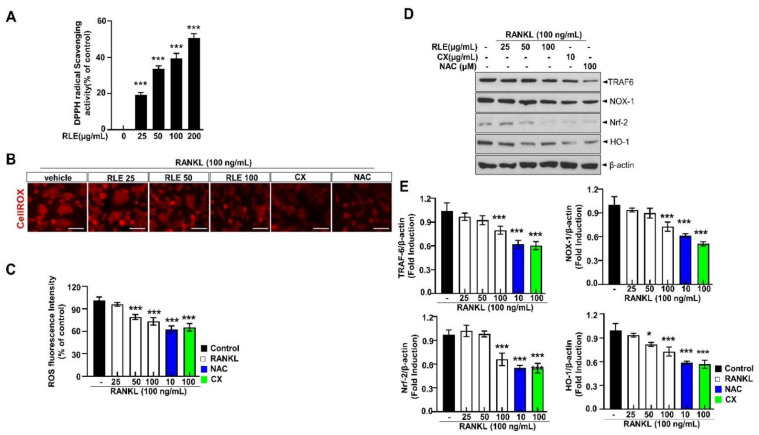
RLE reduces RANKL-induced ROS and its associated signalings during osteoclastogenesis. (**A**) DPPH scavenging activity of RLE. (**B**) Detection of ROS and RANKL-induced osteoclast differentiation in vitro, respectively (scale bar = 100 μm). (**C**) Quantitative analysis of CellROX^®^ Red fluorescence intensity. (**D**) NOX-1 expression. (**E**) Immunoblotting using antibodies against TRAF-6, NOX-1, Nrf-2, HO-1, and β-actin. Values are presented as mean ± SEM. (*n* = 3, * p < 0.05, *** p < 0.001 vs. RANKL) NOX-1, NADPH oxidase 1; CX, canthaxanthin; NAC, N-acetyl cysteine.

RANKL-dependent osteoclast differentiation is largely determined by ROS and could be a therapeutic target for osteolysis treatment. 2,2-diphenyl-1-picrylhydrazyl (DPPH) free radical scavenging is an accepted mechanism for screening the antioxidant activity of plant extracts. RLE dose-dependently regulated DPPH scavenging activity (Figure 8A). Further, CellROX^®^ Red fluorescence was substantially elevated in the RANKL-stimulated group compared to the control group. However, the impact of RANKL was reduced with RLE (Figure 8B,C). Similarly, the expression of NOX-1 was also inhibited in the presence of RLE, canthaxanthin (CX), or N-acetylcysteine (NAC). Further, RANKL binds to its receptor RANK and produces intracellular ROS by activating TRAF-6, NOX-1, Nrf-2, and HO-1 during osteoclastogenesis. TRAF-6, NOX-1, Nrf-2, and HO-1 expressions were significantly upregulated by RANKL stimulation, but RLE dose-dependently regulated these gene expressions (Figure 8D,E). Collectively, these findings indicated the impact of RLE in reducing RANKL-induced ROS production and its directly related signalings. Similarly, it reduces TRAF-6, NOX-1, Nrf-2, and HO-1 and their adaptive signalings during osteoclastogenesis.

## 4. Discussion

The study demonstrates the efficacy of RLE in regulating bone loss in OVX-induced rats. Moreover, RLE inhibited osteoclast differentiation in vitro by preventing osteoclastogenesis through NFATc1/c-Fos downregulation. Moreover, RLE treatment is indicated to improve BMD, bone microarchitecture, and biochemical properties in OVX-induced osteoporosis rats. These study observations suggest RLE as a reliable candidate to control bone remodeling in an osteoporosis state.

OVX is a common method of inducing menopause in rats that reduces estrogen levels in serum. Hence, it is a prominent animal model for investigating female osteoporosis [24]. Several studies have demonstrated that a decrease in estrogen secretion increases dietary intake and white adipose tissue, resulting in metabolic obesity. Generally, osteoporosis is associated with body weight gain. In this study, RLE administration prevented OVX-induced weight gain (Figure 2A,B). However, the uterine size and weight were not affected by the RLE, which was different from estrogen (Figure 2C,D). The administration of RLE restored TV, BV, BV/TV ratio, and trabecular bone loss (Figure 3B–F). These findings strongly indicate that RLE could be a viable alternative treatment for estrogen deficiency-related osteoporosis.

In this model of OVX-induced osteoporosis, RLE inhibits ROS that play a crucial role in the recovery of bone homeostasis. Imbalance in the mechanisms involved in oxidation that generates ROS and antioxidant mechanisms that scavenge ROS contribute to osteoporosis. Thus, oxidative stress is considered critical for the pathogenesis of osteoporosis [25,26]. Reports have shown that ramie leaves are a rich source of various bioactive compounds, including xanthins, benzoic acid, 4-coumaric acid, caffeic acid, ferulic acid, rutin, chlorogenic acid, etc. [6]. All of these compounds have remarkable biological properties, including antioxidant effects. Especially, carotenoids including canthaxanthin have been reported to have antioxidant properties [27,28,29]

In this study, canthaxanthin was selected as a reference compound for standardization of RLE (Figure 1). Expectedly, RLE showed antioxidant effects throughout this study. In addition, RLE increased femoral density and prevented microarchitectural deterioration in OVX rats (Figure 3 and Figure 5A–D), in which ROS and its associated signalings are involved (Figure 6). Furthermore, the study revealed the significance of ROS and NFATC1 pathways in osteoclast differentiation and their function. Induction of RANKL enhanced intracellular ROS levels along with activation of NFATC1 signaling pathways. However, these effects were significantly reduced by RLE and cryptoxanthin, a major component in the RLE (Figure 5E and Figure 6).

RANKL binds to its receptor RANK and recruits TRAF6, which subsequently activates NFATc1, MAPKs, and NF-κB. The activation of these compounds is considered crucial for RANKL-induced osteoclast differentiation. Moreover, TRAF6 indirectly contributes to ROS generation, and its dominant-mutant form reduces intracellular ROS production [30,31]. In addition, Zhou et al. reported that RANKL stimulation increases intracellular ROS [32]. Multiple reports suggest ROS as an upstream molecule that enhances the transcription of osteoclast-specific genes during the inception of RANKL-induced osteoclast formation [17,23]. Meanwhile, plasma-membrane-linked NOX proteins produce ROS, which determines the biological processes. NOX family proteins channel electrons from NADPH to O^2^ when RANKL binds to RANK to produce ROS. This process requires regulatory proteins such as Rac1 [5,33]. However, the function of NOX isoform in osteoclast differentiation is still debated. Among NOX family proteins, NOX-1 influences osteoclast differentiation by interacting with RANKL-RANK signaling. The osteoclastogenesis in vitro data showed that ROS production by RANKL was reduced in the presence of RLE. Further, the effects of NAC on RANKL reveal that RANKL induces ROS production and osteoclast differentiation (Figure 6D and Figure 8B–F). These findings suggest that RLE acts as a ROS scavenger or ROS inhibitor to suppress osteoclast differentiation.

In the present study, RLE inhibited osteoclast activity and osteoclastogenesis. Aberrant bone resorption by osteoclasts influences osteoporosis. Therefore, regulation of osteoclast differentiation could be a novel therapeutic approach to treat osteoporosis. RANKL and macrophage colony-stimulating factors (M-CSF) initiate osteoclastogenesis [20]. RANK and c-Fos may bind to osteoclast precursor surface receptors and activate multiple key transcription factors. Target proteins such as NFATc1 and c-Fos influence osteoclast formation in response to RANKL stimulation, while c-Fos is a key marker for NFATc1 activation [34]. Genes such as cathepsin K, MMP-9, and TRAP contribute to osteoclast differentiation, fusion, and activation [35]. NFATc1 regulates these genes and clarifies the role of RLE in regulating TRAP, cathepsin K, MMP-9, Cal-R, and bone resorption (Figure 7C,D). Furthermore, the expression of osteoclastogenesis marker proteins such as NFATc1, TRAP, c-Fos, and cathepsin K decreased upon RLE supplementation (Figure 7A,B,E).

The study has a few limitations. First, in vivo association between RLE and its predictive active compound has not been thoroughly examined. If the serum level of CX had been evaluated during the pharmacokinetic trial, then the concentrations used in this investigation would have adequately supported the findings. However, the observed concentration of CX in RLE may be adequate to address this specific limitation. Secondly, the correlation between bone biology and endocrine factors has not been investigated. This correlation could be significant if we consider the bone as the target organ for different endocrine factors. Regarding these limitations, further investigation is recommended.

## 5. Conclusions and Future Directions

In conclusion, the investigation findings demonstrate that RLE suppresses RANKL-mediated osteoclast formation by inhibiting NFATc1 and c-Fos expression, which ultimately reduces the expression of osteoclast-specific marker genes. The RLE also decreased RANKL-induced ROS production and its associated signalings, including TRAF-6, NOX-1, Nrf-2, and HO-1. Taken together, our study indicates that RLE can inhibit RANKL-induced osteoclast formation and prevent bone loss in ovariectomized rats. The ROS-inhibiting abilities of RLE are the defining characteristic of RLE against osteoporosis. Therefore, RLE could be a promising therapeutic functional food for postmenopausal osteoporosis. Hence, further comprehensive studies are required to investigate the role of RLE as a therapeutic functional food against osteoporosis and associated disorders.

## Figures and Tables

**Table 1:** RT-PCR primer sequences.

Target Genes	Primer Sequence	Accession Number
Cathepsin K	F: 5′-atc tct ctg tac cct ctg ca-3′ R: 5′-cct ctc ttg gtg tcc ata ca-3′	NM_007802.4
Cal-R	F: 5′-tgc att ccc ggg ata cac ag-3′ R: 5′-agg aac gca gac ttc act gg-3′	NM_001355192.1
TRAP	F: 5′-act tcc cca gcc ctt act acc g-3′ R: 5′-tca gca cat agc cca cac cg-3′	NM_007388.3
MMP-9	F: 5′-cga ctt ttg tgg tct tcc cc-3′ R: 5′-tga agg ttt gga atc gac cc-3′	NM_013599.4
GAPDH	F: 5′-act ttg tca agc tca ttt cc-3′ R: 5′-tgc agc gaa ctt tat tga tg-3′	NM_008084.3
